# Sigma-1 Receptor Modulation by Ligands Coordinates Cancer Cell Energy Metabolism

**DOI:** 10.3390/biom12060762

**Published:** 2022-05-30

**Authors:** Furkan E. Oflaz, Zhanat Koshenov, Martin Hirtl, Rene Rost, Roland Malli, Wolfgang F. Graier

**Affiliations:** 1 Molecular Biology and Biochemistry, Gottfried Schatz Research Center, Medical University of Graz, 8010 Graz, Austria; furkan.oflaz@medunigraz.at (F.E.O.); zhanat.koshenov@medunigraz.at (Z.K.); martin.hirtl@medunigraz.at (M.H.); rene.rost@medunigraz.at (R.R.); roland.malli@medunigraz.at (R.M.); 2 BioTechMed Graz, 8010 Graz, Austria

**Keywords:** sigma-1 receptor, cancer metabolism, mitochondrial bioenergetics, A549, MCF7

## Abstract

Sigma-1 receptor (S1R) is an important endoplasmic reticulum chaperone with various functions in health and disease. The purpose of the current work was to elucidate the involvement of S1R in cancer energy metabolism under its basal, activated, and inactivated states. For this, two cancer cell lines that differentially express S1R were treated with S1R agonist, (+)-SKF10047, and antagonist, BD1047. The effects of the agonist and antagonist on cancer energy metabolism were studied using single-cell fluorescence microscopy analysis of real-time ion and metabolite fluxes. Our experiments revealed that S1R activation by agonist increases mitochondrial bioenergetics of cancer cells while decreasing their reliance on aerobic glycolysis. S1R antagonist did not have a major impact on mitochondrial bioenergetics of tested cell lines but increased aerobic glycolysis of S1R expressing cancer cell line. Our findings suggest that S1R plays an important role in cancer energy metabolism and that S1R ligands can serve as tools to modulate it.

## 1. Introduction

Sigma-1 Receptor (S1R) is an integral membrane protein localized to the endoplasmic reticulum (ER) and highly enriched in specialized regions named mitochondria-associated ER membranes (MAMs) [1]. S1R is abundant in the Central Nervous System (CNS), but it is also highly expressed in liver, lung and different cancer cells [2]. Under basal state, S1R was shown to interact with ER-resident chaperone BiP [1,3]. Upon ER stress or agonist stimulation, S1R dissociates from BiP and interacts with many of its target proteins, including Inositol 1,4,5-trisphosphate receptors (IP_3_R) [1]. Through the latter interaction, S1R can regulate efficient Ca^2+^ delivery to mitochondria. Moreover, agonist activation of S1R can lead to translocation of S1R to the cell membrane, where it regulates the activity of ion channels [4]. Consequently, S1R plays an important role in ion channel activity [4], apoptosis [5], and proliferation [6]. 

Localizing in different integral membranes, interacting with diverse classes of proteins, and activation-dependent regulation of different cellular pathways make S1R a potential target for metabolic modulation of cancer cells. S1R is highly expressed in several cancers including prostate, colon, melanoma, breast, and lung [7,8]. Several studies investigated the effects of S1R ligands in different cancer cell lines [5,6,7,8,9]. Among lung, breast, and prostate cancer cell lines, the highest expression of S1R was found in lung then breast, and colon cancer cell lines [6]. In these studies, an agonist of S1R, SKF10047, reduced the proliferation of high S1R expressing MDA-MB-231 breast cancer cell line but not of MCF-7 or MCF-10A cell lines, which have low levels of S1R expression [6]. Moreover, the S1R antagonist, Rimcazole, promotes caspase-dependent apoptosis in cancer cells and this effect was attenuated by S1R agonist SKF10047 [5]. Altogether these studies suggest that S1R expression, ligand activation, and function can determine cancer cell fate. 

In this study, we investigated the role of S1R under basal, activated, and inactivated states on cancer energy metabolism by live-cell imaging of metabolic fluxes and ions in mitochondria and cytosol. Our results reveal that pharmacological activation of S1R increases mitochondrial bioenergetics by increasing basal Ca^2+^ delivery to mitochondrial matrix in highly S1R expressing A549 cells.

## 2. Materials and Methods

### 2.1. Cell Culture and Transfection

A-549 and MCF-7 were bought from the cell culture facility of the Medical University of Graz. Cells were cultivated in DMEM (D5523, Sigma-Aldrich, Vienna, Austria) with 10% FCS (Gibco, Life Technologies, Vienna, Austria) in a humidified incubator (37 °C, 5% CO_2_, 95% air). Both cell lines were authenticated and mycoplasma free. For microscopic measurements, 1 to 2 days before the transfection A549 and MCF7 cells were plated on 30 mm glass coverslips. After reaching 40–50% confluency cells were transfected with different plasmids such as S1R-mCherry (1 µg/well), mt.AT1.03 (0.6 µg/well) [10], Lapronic (0.6 µg/well) [11], 4mtD3cpv (0.8 µg/well) [12] and D3cpv (0.8 µg/well) [12] with or without 160 nM/well S1R siRNA (siRNA sequence: 5′-GCU CAC CAC CUA CCU CUU UdTdT-3′). Both cell lines were transfected with TransFast transfection reagent, 3 µL/well, (Promega, Madison, WI, USA) in 1 mL serum and antibiotic free-medium. 12 to 14 h after the transfection, transfection media was replaced with 2 mL of DMEM media. All control A549 cells in experiments with siRNA against S1R were transfected with scrambled siRNA. Cells were treated with S1R ligands at final concentration of 10 µM 2–4 h before measurements in DMEM and kept in humidified incubator. 

### 2.2. Buffers and Solutions

Prior to microscopic measurements, cells were adjusted to room temperature for 10–12 min in storage buffer: 2 mM Ca^2+^, 138 mM NaCl, 1 mM MgCl_2_, 5 mM KCl, 10 mM HEPES, 2.6 mM NaHCO_3_, 0.44 mM KH2PO4, amino acid and vitamin mix, 10 mM glucose, 2 mM L-glutamine, 1% penicillin/streptomycin, 1.25 μg/mL amphotericin B and pH adjusted to 7.4. Respective S1R ligands were present in storage buffers. All live-cell imaging experiments were performed in the experimental buffer: 2 mM CaCl_2_, 138 mM NaCl, 1 mM MgCl_2_, 5 mM KCl, 10 mM Hepes, and 10 mM D-glucose at pH 7.4. For glucose deprivation experiments we used the same buffer without glucose: 2 mM CaCl_2_, 138 mM NaCl, 1 mM MgCl_2_, 5 mM KCl, and 10 mM Hepes at pH 7.4. SKF10047 and BD1047 were obtained from Tocris (Tocris, Abingdon, UK) and dissolved in water to prepare 10 mM stock solutions.

### 2.3. Western Blot

Cells were seeded in 6 well plates and transfected with either Control or S1R siRNA as described above. After 2 days the cells were harvested in RIPA buffer (25 mM Tris-HCl pH 7.6, 150 mM NaCl, 5 mM EDTA, 1% Triton X-100, 1% sodium deoxycholate, 0.1% SDS) supplemented with protease inhibitor cocktail (#P8340 Sigma, Vienna, Austria). Proteins were extracted and concentration was determined by Pierce™ BCA Protein Assay Kit (ThermoFisher Scientific, Waltham, MA, USA) on a CLARIOstar Plus (BMG Labtech, Ortenberg, Germany). The concentration-adjusted samples were resolved on a 12.5% SDS-PAGE at a constant 120 V and subsequently transferred to a Immobilon-P membrane (PVDF, 0.45 µm, Merck, Vienna, Austria). Membranes were incubated at 4 °C in primary antibodies overnight, washed in 1xTBS-T, and then incubated in the corresponding secondary antibody for 1h at room temperature. In the end, SuperSignalTM West Pico PLUS Chemiluminescent Substrate (ThermoFisher Scientific) was applied to the membranes, the signal was captured on a ChemiDoc MP Imaging System (Biorad) and quantified via image-J Fiji [13]. List of used protein ladder and antibodies: SIGMAR1 (D4J2E) Rabbit mAb #61994 (Cell Signaling); β-Actin (D6A8) Rabbit mAb #8457 (Cell Signaling); goat anti-rabbit IgG-HRP: sc-2054 (Santa Cruz Biotechnology, Dallas, TX, USA); Color Prestained Protein Standard, Broad Range (10–250 kDa) (P7719S, New England BioLabs, Waltham, MA, USA).

### 2.4. Live-Cell Imaging Experiments

Experiments were performed with an Olympus IX73 inverted microscope that is equipped with an UApoN340 40× oil immersion objective (Olympus, Tokyo, Japan) and a CCD Retiga R1 camera (Q-imaging, Surrey, BC, Canada). For illumination, LedHUB^®^ (Omnicron, Germany) equipped with 340, 385, 455, 470, and 550 nm LEDs in combination with CFP/YFP/RFP (CFP/YFP/mCherry-3X, Semrock, New York, NY, USA) filter set was used. Visiview 4.2.01 (Visitron, Puchheim, Germany) was used for the data acquisition. Alternatively, an AnglerFish F-G/O (Next Generation Fluorescence Imaging/NGFI (www.ngfi.eu, accessed on 16 May 2022), Graz, Austria) was used for data acquisition. During the measurements, cells were perfused by a gravity-based perfusion system PS-9D (NGFI, Graz, Austria). Briefly, nine positions of the valve are connected with reservoirs and the reservoir of interest can be automatically activated via perfusion control software. The flow rate of the reservoir manually adjusted to 1 ml/min. All the experiments were done at room temperature without specific temperature or gas control. Cells were chosen randomly based on the expression of respective genetically encoded biosensors, and in the case of mitochondrial-targeted probes, based on correct localization. 

### 2.5. Mitochondrial ATP Measurements

Mitochondrial ATP was measured using genetically encoded, FRET-based, mitochondrial matrix targeted ATP sensor mt.AT1.03 [10] (gift from Hiromi Imamura, Kyoto University, Kyodai Graduate School of Biostudies, Japan). mt.AT1.03 consist of ε subunit of the bacterial FoF1-ATP synthase sandwiched in between cyan and yellow fluorescent proteins (FP). Upon ATP binding to the ε subunit of the bacterial FoF1-ATP synthase, it goes to conformational change, which affects the distance between the cyan and yellow fluorescent proteins. This change affects the emission of the FRET pair and gives the ratiometric readout of the mitochondrial ATP changes. This sensor was excited by 455 nm LED with a 300-millisecond exposure time every 2 s and emission was collected at 480 nm and 530 nm using a CFP/YFP/mCherry-3X filter set and 505dcxr beam-splitter. Background subtracted emission ratio of 530/480 was analyzed.

After adjusting cells in loading buffer for 10 min, cells were perfused in experimental buffer and basal mitochondrial ATP levels were measured for 2 min. To obtain mitochondrial ATP response upon glucose deprivation and oligomycin treatment the buffer was changed to an experimental buffer without glucose for 8 min. At the end of 8 min, cells were perfused for 4 min in glucose-containing buffer and an additional 6 min with 2 µM oligomycin. Oxphos/Glycolysis ratio was obtained by the ratio change after oligomycin addition divided by the change in ratio upon glucose deprivation.

### 2.6. Mitochondrial and Cytosolic Ca^2+^ Measurements

Mitochondrial and Cytoplasmic Ca^2+^ were measured using genetically encoded, FRET-based, mitochondria and cytoplasm targeted Ca^2+^ sensors 4mtD3cpv and D3cpv, respectively [12]. 4mtD3cpv and D3cpv consist of mammalian calmodulin (CaM) and M13 peptide of myosin light chain kinase (CaM/M13) sandwiched in between cyan and yellow FP. Upon Ca^2+^ binding to CaM, it undergoes conformational change via binding with the M13 peptide. This change affects the emission of the FRET pair and gives us the ratiometric readout of the mitochondrial and cytoplasmic Ca^2+^ changes. Each sensor was excited with a 455 nm LED for every 3 s with a 300-millisecond exposure time and emission was collected at 480 nm and 530 nm using a CFP/YFP/mCherry-3X filter set and 505dcxr beam-splitter. Background subtracted emission ratio 530/480 was analyzed. Briefly, cells were adjusted in a storage buffer for 10 min. Next, cells were perfused in an experimental buffer and either basal mitochondrial or cytoplasmic Ca^2+^ ratios were measured from several different cells. Next, to obtain ATP-induced maximum mitochondrial or cytoplasmic Ca^2+^ rise, cells were stimulated with 100 µM ATP containing experimental buffer.

### 2.7. Mitochondrial Membrane Potential Measurements

Mitochondrial membrane potential experiments were done by using tetramethylrhodamine methyl ester perchlorate (TMRM) dye. TMRM was excited with 550 nm LED for every 2 s with 300-millisecond exposure time and emission was collected at 600 nm using a CFP/YFP/mCherry-3X filter set. Briefly, cells were incubated for 20 min in a storage buffer containing 25 nM of TMRM. After incubation, cells were perfused in the experimental buffer for 1 min and the basal value was recorded. After baseline recording cells were perfused 1 µM FCCP containing experimental buffer to fully depolarize the mitochondria. Background subtracted mitochondria to nucleus ratio was used as a readout.

### 2.8. Cytosolic Pyruvate/Lactate Measurements

Cytosolic pyruvate to lactate ratio was measured using genetically encoded, FRET-based, cytosol targeted sensor named Lapronic (AddGene, #140756) [11]. Lapronic consists of transcriptional factor LutR (which can bind both to pyruvate and lactate) of Bacillus licheniformis sandwiched in between cyan and yellow fluorescent proteins (FP). Upon pyruvate or lactate binding to the LutR, LutR goes to conformational change. This change affects the emission of the FRET pair and gives us the ratiometric readout of the cytoplasmic pyruvate and lactate changes. This sensor was excited with 455 nm LED for every 2 s with 300-millisecond exposure time and emission was collected at 480 nm and 530 nm using a CFP/YFP/mCherry-3X filter set and 505dcxr beam-splitter. Background subtracted emission ratio 530/480 was analyzed. Briefly, after adjusting cells in storage buffer for 10 min, cells were perfused in experimental buffer and basal pyruvate/lactate ratio was measured.

### 2.9. Data Analysis

Data shown were acquired from a minimum three different days and represent the mean ± SEM. The number of independent experiments and the cell number were represented as “*n* = single-cell/independent experiment” in figure legends. Single cells were used for the statistical analysis, where analysis of variance (ANOVA) with Tukey post hoc test was performed. GraphPad Prism software version 9.3.1 (GraphPad Software, San Diego, CA, USA), Microsoft Excel (Microsoft), were used for the analysis, calculation, and representation of the data. Representative images were analyzed using Image-J freeware program (NIH, Waltham, MA, USA) [13].

## 3. Results

### 3.1. Sigma-1 Receptor Is Differentially Expressed in A549, Lung Carcinoma, and MCF7, Breast Cancer, Cell Lines

**Figure 1:**
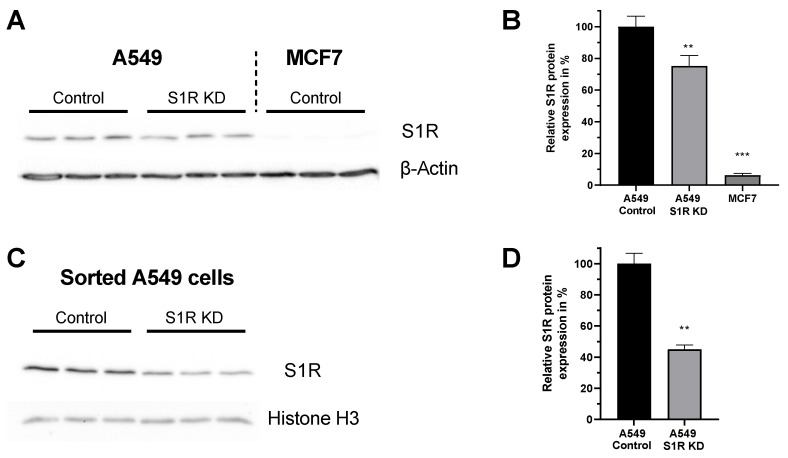
Expression level of S1R in A549 and MCF-7 Cells. (**A**) Immunoblot images show the expression level of S1R in cells transfected either with control siRNA or siRNA against S1R (left) in A549 cells and non-transfected MCF7 cells (right). (**B**) Bar graphs represent immunoblot analysis of S1R expression as MEAN±SEM in A549 cells transfected with control siRNA or siRNA against S1R and non-transfected MCF7 cells. (**C**) Immunoblot images show the expression level of S1R in cells transfected either with control siRNA or siRNA against S1R in sorted, transfection positive A549 cells. (**D**) Bar graphs represent immunoblot analysis of S1R expression as MEAN±SEM in sorted A549 cells. Significant differences were assessed using one-way ANOVA with Tukey’s multiple comparison test (for **B**) or unpaired t-test (for **D**) and presented as (** *p* < 0.01, *** *p* < 0.001). A549 cells: A549 Control (*n* = 3), A549 S1R KD (*n* = 3), MCF7: (*n* = 3), sorted A549 Control (*n* = 3), sorted A549 S1R KD (*n* = 3).

For this study we have chosen two human cancer cell lines that differentially express S1R protein [14]. A549 lung adenocarcinoma cell line expresses S1R, while MCF7 breast cancer cell line expresses barely detectable levels of S1R (Figure 1A,B), and thus serves as a good control cell line. Additionally, the knockdown (KD) of S1R in A549 cells using small interfering RNA (siRNA) resulted in a 25% reduction of S1R protein level when analyzed without transfection selection marker (Figure 1B). As we have recently shown, this knockdown efficiency does not reflect the extent of protein downregulation in positively transfected single cells [15]. Hence, we have quantified protein downregulation in transfection positive sorted A549 cells and saw a 55% downregulation of S1R protein (Figure 1C,D). Since overall transfection efficiency of both cell lines using our transfection protocol is around 30–40%, we have relied on single-cell experiments with transfection selection markers for experiments with S1R KD.

### 3.2. Pharmacological Activation of S1R Increases Mitochondrial Bioenergetics While the Antagonist Doesn’t Affect It

**Figure 2:**
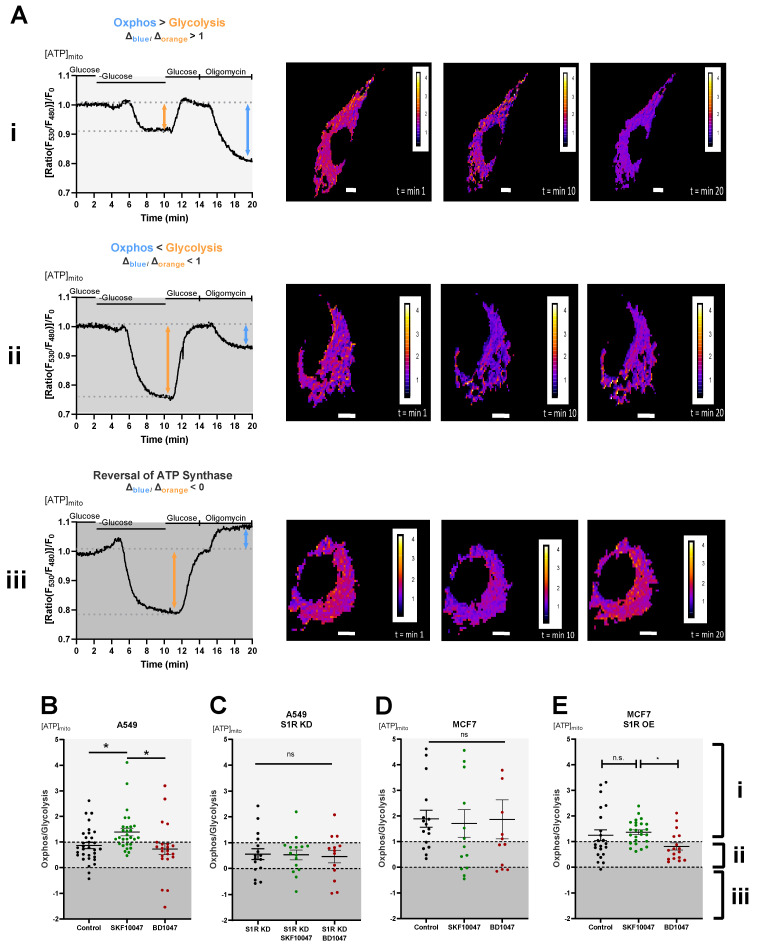
Effect of pharmacological activation of S1R on mitochondrial energy status. (**A**) Representative traces of mitochondrial ATP levels with corresponding representative images of the cells transfected with mtAT.1.03 in MCF7 (**i**) and A549 (**ii**,**iii**) cells. The cells have been pseudocolored to represent mitochondrial ATP level as mtAT1.04 ratio; calibration bars are inserted on the right-hand side of the cells. The inserted white scale bar represents 10 µm. Change in ratio of oligomycin addition to glucose deprivation was used as a Oxphos/Glycolysis ratio and presented as Oxphos > Glycolysis (**A**(**i**)), Oxphos < Glycolysis (**A**(**ii**)), and reversal of ATP synthase (**A**(**iii**)). White area in panels (**B**–**E**) (indicated with (**i**)) represents Oxphos > Glycolysis, light gray area in panels (**B**–**E**) (indicates with (**ii**)) represents Oxphos < Glycolysis, and dark gray area in panels (**B**–**E)** (indicated by (**iii**)) represents reversal of ATP synthase. Scatter plots with individual values represent the ratio of Oxphos to Glycolysis in single cells with MEAN±SEM for (**B**) Control (black), Control+SKF10047 (green), Control+BD1047 (red), (**C**) S1R KD (black), S1R KD +SKF10047 (green) and S1R KD +BD1047 (red) in A549 cells, (**D**) for Control (black), Control+SKF10047 (green), Control+BD1047 (red) in MCF7 cells, and (**E**) for Control (black), Control+SKF10047 (green), Control+BD1047 (red) in MCF7 cells with S1R OE. Cells were treated with BD1047 and SKF10047 2–4 h prior to each experiment. Significant differences were assessed using one-way ANOVA with Tukey’s multiple comparison test and presented as (* *p* < 0.05, ns: not significant). A549 cells: Control (32 cells/16 experiments), Control + SKF10047 (29 cells/15 experiments), Control + BD1047 (22 cells/13 experiments), SR1 KD (16 cells/10 experiments), S1R KD + SKF10047 (15 cells/10 experiments) and S1RKD + BD1047 (13 cells/8 experiments). MCF7 cells: Control (15 cells/6 experiments), Control + SKF10047 (13 cells/4 experiments), Control + BD1047 (11 cells/5 experiments), S1R OE (22 cells/7 experiments), S1R OE + SKF10047 (25 cells/8 experiments), and S1R OE + BD1047 (18 cells/5 experiments).

To dissect the roles of S1R in cellular energy metabolism under basal, activated, and inactivated states we have performed real-time mitochondrial ATP measurements using genetically encoded mitochondrial ATP biosensor mtAT1.03 [10] (Figure 2A(i–iii)). We have deployed a protocol previously published by our group [16,17], which consists of two main steps: first, the glucose removal step, gives information on the reliance of the cells on glucose metabolism and mainly reflects glycolytic ATP flux, while the second step, oligomycin addition following re-introduction of glucose, reflects mitochondrial ATP production, thus oxidative phosphorylation (OXPHOS) level. The ratio of the second (oligomycin addition) to the first (glucose removal) step reflects the metabolic state of a cell and its reliance on OXPHOS versus glycolysis (Figure 2A(i–iii)). Activation of S1R using its well-described agonist, SKF-10047 [18], resulted in enhanced mitochondrial bioenergetics of A549 cells and increased their reliance on mitochondrial versus glycolytic ATP production (Figure 2B). The application of the specific S1R antagonist, BD-1047 [19], did not have a major effect on the metabolic state and mitochondrial bioenergetics of these cells (Figure 2B). The effect of (+)-SKF-10047 was abolished by the KD of S1R in A549 cells (Figure 2C) and neither S1R agonist nor antagonist had any effect in MCF7 cells (Figure 2D), suggesting S1R specificity of activation by (+)-SKF-10047. These findings point at OXPHOS promoting action of pharmacological S1R activation, while inactivation does not yield much of an effect, implying that S1R is mostly in its inactive form under the resting condition in regards to cellular energy metabolism. 

To further validate these results, we have overexpressed (OE) S1R in MCF7 cells (Supplementary Figure S1), and performed the same protocol. Although the OE of S1R-mCherry construct altered overall metabolic response of the MCF7 cells, (+)-SKF-10047 had a clear OXPHOS promoting effect in comparison to BD1047 treated cells (Figure 2E).

In contrast to oligomycin addition glucose deprivation ratio readout, analysis of basal ATP level did not provide a significant difference upon agonist or antagonist treatment (Supplementary Figure S2). 

### 3.3. S1R Agonist Increases Mitochondrial Membrane Potential and Reduces Aerobic Glycolysis, While the Antagonist Increases Aerobic Glycolysis 

**Figure 3:**
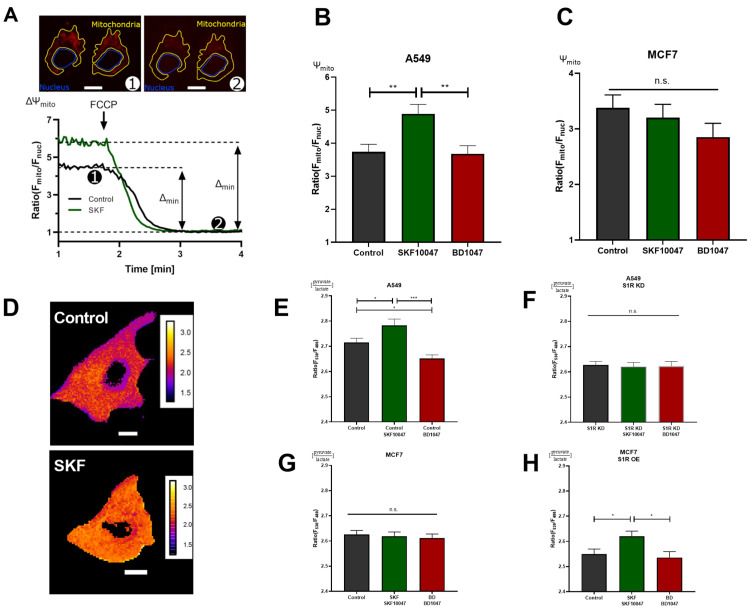
S1R ligands modulate cancer cell energy metabolism. (**A**) Exemplary images of A549 cells stained with TMRM and selected regions of interest (ROI) for mitochondria and nucleus. The inserted white scale bar represents 10 µm. The experimental protocol with representative ratio traces of mitochondrial to nucleus ROIs is shown for control (black) and control+SKF10047 (green) are shown. Protocol indicated in (**A**) was used to obtain mitochondrial membrane potential for Control (black) and Control+SKF10047 (green) by calculating the change in fluorescence ratio in mitochondria and in nucleus after 1 µM FCCP treatment. (**B**) Bar graphs with MEAN±SEM represent the mitochondrial membrane potential for Control (black), Control+SKF10047 (green) and Control+BD1047 (red) in A549 cells and (**C**) in MCF7 cells. (**D**) Exemplary images of A549 cells transfected with ratiometric pyruvate/lactate sensor for Control (above) and Control+SKF10047 (below). The cells have been pseudocolored to represent pyruvate/lactate ratio, calibration bars are inserted on the right-hand side of both cells. The inserted white scale bar represents 10 µm. (**E**) Bar graphs with MEAN±SEM represent the cytoplasmic ratio of pyruvate to lactate for Control (black), Control+SKF10047 (green), Control+BD1047 (red), (**F**) S1R KD (black), S1R KD +SKF10047 (green) and S1R KD +BD1047 (red) in A549 cells, (**G**) Control (black), Control+SKF10047 (green), Control+BD1047 (red) in MCF7 cells, and (**H**) Control (black), Control+SKF10047 (green), Control+BD1047 (red) in MCF7 cells with S1R OE. Cells were treated with BD1047 and SKF10047 2–4 h prior to each experiment. Significant differences were assessed using one-way ANOVA with Tukey’s multiple comparison test and presented as (* *p* < 0.05, ** *p* < 0.01, *** *p* < 0.001, ns: not significant). A549 cells TMRM measurements: Control (90 cells/4 experiments), Control + SKF10047 (114 cells/5 experiments), Control + BD1047 (92 cells/4 experiments). MCF7 cells TMRM measurements: Control (72 cells/4 experiments), Control + SKF10047 (111 cells/4 experiments) and Control + BD1047 (81 cells/4 experiments). A549 cells pyruvate to lactate ratio: Control (69 cells/6 experiments), Control + SKF10047 (60 cells/6 experiments), Control + BD1047 (69 cells/6 experiments), SR1 KD (58 cells/5 experiments), S1R KD + SKF10047 (49 cells/4 experiments) and S1RKD + BD1047 (45 cells/4 experiments). MCF7 cells pyruvate to lactate ratio: Control (54 cells/4 experiments), Control + SKF10047 (52 cells/4 experiments) and Control + BD1047 (53 cells/4 experiments). S1R-mCherry OE in MCF7 cells pyruvate to lactate ratio: Control (65 cells/8 experiments), Control + SKF10047 (83 cells/9 experiments) and Control + BD1047 (49 cells/7 experiments).

To further corroborate the results obtained with real-time mitochondrial ATP measurements without transfecting the cells, we deployed mitochondrial membrane potential (Ψ_m_) assessment with a tetramethylrhodamine methyl ester (TMRM), a cationic fluorescent dye that accumulates in mitochondria depending on membrane potential (Figure 3A). In support of the previous experiment, activation of S1R with (+)-SKF10047 increased, while BD1047 did not affect Ψ_m_ in A549 cells (Figure 3B). Similar to ATP experiment, neither of the compounds changed Ψ_m_ in MCF7 cells (Figure 3C). As Ψ_m_ is a reflection of mitochondrial energetic status and directly represents cellular OXPHOS, S1R has an important role in promoting OXPHOS upon activation, while under basal state, it seems to be dormant in regards to this particular function, since there was no effect upon antagonist application. 

Next, we assessed the cytosolic pyruvate/lactate ratio with genetically encoded sensor Lapronic [11] (Figure 3D). Activation of S1R with the agonist resulted in increased pyruvate/lactate ratio in A549 cells and in MCF7 cells with S1R OE, but not in the case of S1R KD or MCF7 cells without OE (Figure 3 E–H). Interestingly, the antagonist, as well as S1R KD, reduced the pyruvate/lactate ratio in A549 cells (Figure 3E–G). Pyruvate/lactate ratio can be used to deduce the cell’s reliance on aerobic glycolysis versus Oxphos, as the increase of the ratio ((+)-SKF10047 in A549 and MCF7 with S1R OE) would suggest the cell is producing less lactate relative to pyruvate, and likely rerouting the pyruvate towards tricarboxylic acid (TCA) cycle. In case of decreased pyruvate/lactate ratio (BD1047 and S1R KD in A549 cells), the cell contains more lactate relative to pyruvate, suggesting it is performing more aerobic glycolysis. The pyruvate/lactate ratio measurements added a new perspective on the action of S1R under its basal, activated, and inactivated states. We have already shown that activation of S1R promotes OXPHOS. Based on pyruvate/lactate ratio, it also reduces aerobic glycolysis, while inactivation or KD of S1R seems to increase aerobic glycolysis, while not affecting mitochondrial ATP production or Ψ_m_, suggesting basal involvement of S1R in balancing aerobic glycolysis and OXPHOS.

### 3.4. S1R Activation Boosts Mitochondrial Ca^2+^ Homeostasis 

**Figure 4:**
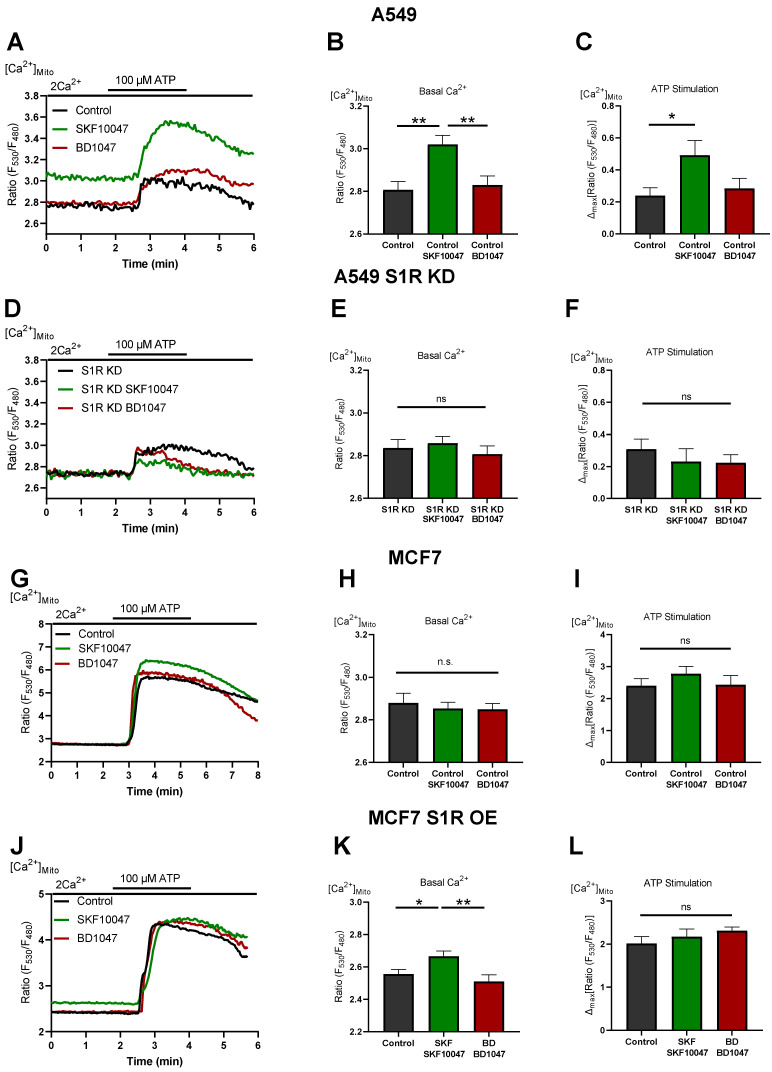
S1R activation increases basal and agonist-induced mitochondrial Ca^2+^ uptake. (**A**) Representative traces of mitochondrial Ca^2+^ dynamics measured with 4mtD3cpv for control (black), Control+SKF10047 (green) and Control + BD1047 (red) in A549 cells. (**B**) Bar graphs with MEAN±SEM represent basal mitochondrial Ca^2+^ level for Control (black), Control+SKF10047 (green) and Control+BD1047 (red) in A549 cells. (**C**) Bar graphs with MEAN±SEM represent ATP (100 µM) induced mitochondrial Ca^2+^ uptake for Control (black), Control+SKF10047 (green) and Control+BD1047 (red) in A549 cells. (**D**) Representative traces of mitochondrial Ca^2+^ dynamics for S1R KD (black), S1R KD +SKF10047 (green) and S1R KD +BD1047 (red) in A549 cells. (**E**) Bar graphs with MEAN±SEM represent basal mitochondrial Ca^2+^ level for S1R KD (black), S1R KD +SKF10047 (green) and S1R KD +BD1047 (red) in A549 cells. (**F**) Bar graphs with MEAN±SEM represent ATP (100 µM) induced mitochondrial Ca^2+^ uptake for S1R KD (black), S1R KD +SKF10047 (green) and S1R KD +BD1047 (red) in A549 cells. (**G**) Representative traces of mitochondrial Ca^2+^ dynamics for Control (black), Control+SKF10047 (green) and Control+BD1047 (red) in MCF7 cells. (**H**) Bar graphs with MEAN±SEM represent basal mitochondrial Ca^2+^ level for Control (black), Control+SKF10047 (green) and Control+BD1047 (red) in MCF7 cells. (**I**) Bar graphs with MEAN±SEM represent ATP (100 µM) induced mitochondrial Ca^2+^ uptake for Control (black), Control+SKF10047 (green) and Control+BD1047 (red) in MCF7 cells. (**J**) Representative traces of mitochondrial Ca^2+^ dynamics for Control (black), Control+SKF10047 (green) and Control+BD1047 (red) in S1R-mCherry OE MCF7 cells. (**K**) Bar graphs with MEAN±SEM represent basal mitochondrial Ca^2+^ level for Control (black), Control+SKF10047 (green) and Control+BD1047 (red) in S1R-mCherry OE MCF7 cells. (**L**) Bar graphs with MEAN±SEM represent ATP (100 µM) induced mitochondrial Ca^2+^ uptake for Control (black), Control+SKF10047 (green) and Control+BD1047 (red) in S1R-mCherry OE MCF7 cells. Cells were treated with BD1047 and SKF10047 2–4 h prior to each experiment. Significant differences were assessed using one-way ANOVA with Tukey’s multiple comparison test and presented as specific *p*-values (* *p* < 0.05, ** *p* < 0.01, ns: not significant). A549 cells basal Ca^2+^ measurements: Control (44 cells/9 experiments), Control + SKF10047 (44 cells/8 experiments), Control + BD1047 (38 cells/7 experiments), SR1 KD (32 cells/7 experiments), S1R KD + SKF10047 (43 cells/8 experiments) and S1RKD + BD1047 (25 cells/7 experiments). A549 cells ATP stimulation: Control (17 cells/9 experiments), Control + SKF10047 (20 cells/8 experiments), Control + BD1047 (18 cells/7 experiments), SR1 KD (11 cells/7 experiments), S1R KD + SKF10047 (12 cells/8 experiments) and S1RKD + BD1047 (9 cells/7 experiments). MCF7 cells basal Ca^2+^ measurements: Control (34 cells/3 experiments), Control + SKF10047 (54 cells/5 experiments) and Control + BD1047 (44 cells/4 experiments). MCF7 cells ATP stimulation: Control (16 cells/3 experiments), Control + SKF10047 (18 cells/5 experiments) and Control + BD1047 (20 cells/4 experiments). S1R-mCherry OE MCF7 cells basal Ca^2+^ measurements: Control (26 cells/6 experiments), Control + SKF10047 (31 cells/6 experiments) and Control + BD1047 (22 cells/6 experiments). S1R-mCherry OE MCF7 cells ATP stimulation: Control (17 cells/6 experiments), Control + SKF10047 (17 cells/6 experiments) and Control + BD1047 (14 cells/6 experiments).

To investigate the mechanism of action of S1R activation and inactivation, we measured mitochondrial Ca^2+^ levels in A549 and MCF7 cells (Figure 4). (+)-SKF10047 treatment increased basal mitochondrial Ca^2+^ level as well as IP_3_ generating agonist-induced mitochondrial Ca^2+^ uptake in control A549 cells (Figure 4A–C). This effect was abolished by S1R KD (Figure 4D–F) and was not present in MCF7 cells (Figure 4G–I). OE of S1R-mCherry construct in MCF7 cells mimicked the increased basal mitochondrial Ca^2+^ level induced by (+)-SKF10047, but not IP_3_ generating agonist-induced mitochondrial Ca^2+^ uptake, which was unaffected (Figure 4J–L). These results provide a mechanistic insight into Oxphos promoting effect of S1R activation, whereby (+)-SKF10047 promotes mitochondrial bioenergetics by increasing basal mitochondrial Ca^2+^ level as well as mitochondrial Ca^2+^ uptake, thus boosting both mitochondrial matrix residing Ca^2+^ sensitive NADH dehydrogenases along with mitochondrial intermembrane space (IMS) residing Ca^2+^ sensitive metabolite shuttles [17,18]. Together, these effects can theoretically overweight depolarizing effect of increased basal Ca^2+^ in mitochondria [20,21]. 

On the other hand, BD1047 had no effect on basal Ca^2+^ level as well as on mitochondrial Ca^2+^ uptake (Figure 4). Hence, mitochondrial Ca^2+^ data do not provide an explanation for increased aerobic glycolysis under S1R inactivation by BD1047 or S1R KD. None of the compounds had a considerable impact on basal or agonist-induced Ca^2+^ increase in the cytosol (Supplementary Figure S3).

## 4. Discussion

It is well known that cancer cells reprogram their metabolism to fit their demand for uncontrolled proliferation and survival [22]. Cancer energy metabolism is targeted by various anticancer treatments [23], but still requires a better understanding to design more efficient treatments strategies. In this study, we wanted to investigate the role of S1R in cancer energy metabolism. In particular, we were interested in basal, activated, and inactivated states of S1R. For this, we have chosen two cancer cell lines that differentially express S1R (Figure 1A,B) and treated them with established S1R agonist (+)-SKF10047 or antagonist BD1047. Additionally, we knocked-down S1R in cells expressing the protein (A549, Figure 1A–D) and overexpressed it in cells that have low levels of S1R (MCF7, Supplementary Figure S1), to test the specificity of the S1R ligands in their action on cancer energy metabolism.

Using real-time mitochondrial ATP, Ψ_m_, and cytosolic pyruvate/lactate ratio measurements, we have established that activation of S1R by its agonist enhances OXPHOS and reduces reliance on aerobic glycolysis in A549 cells and in MCF7 cells transiently overexpressing S1R (Figure 2B,E and Figure 3B,E,H). In contrast, the S1R antagonist and S1R KD did not have an impact on OXPHOS, but increased aerobic glycolysis (Figure 2B,C and Figure 3B,E,F) in A549 cells, suggesting that S1R has a metabolic balancing function. Based on our results and previous publications [15], S1R seems to be rather dormant regarding its influence on mitochondrial bioenergetics under resting conditions, since S1R antagonist and S1R KD did not affect mitochondrial ATP and Ψ_m_ (Figure 2B,C and Figure 3E,F) and S1R OE in MCF7 cells did not drastically change metabolic phenotype of the cells unless treated with the agonist (Figure 2E and Figure 3H). As MCF7 serves as a control cell line with negligible S1R expression, none of the ligands impacted measured parameters in MCF7 cells (Figure 2D and Figure 3C,G). 

We have recently shown that S1R is indispensable for enhancing mitochondrial bioenergetics during early ER stress by orchestrating ER Ca^2+^ leak towards mitochondria [15]. As it is known that ER stress activates S1R, we wanted to test if pharmacological activation of S1R also increases mitochondrial Ca^2+^ levels and thus explains boosted mitochondrial bioenergetics. Indeed, activation of S1R with (+)-SKF10047 increased both basal Ca^2+^ levels as well as mitochondrial Ca^2+^ uptake upon ER Ca^2+^ release in A549 cells (Figure 4A–C), while not affecting cytosolic Ca^2+^ levels (Supplementary Figure S3A–C). This increase of basal Ca^2+^ level and increased ER Ca^2+^ release directed towards mitochondria are likely responsible for increased mitochondrial bioenergetics upon S1R activation, as Ca^2+^ is a known regulator of cellular and mitochondrial bioenergetics [20,24]. Interestingly, (+)-SKF10047 treatment of transiently S1R overexpressing MCF7 cells only increased basal mitochondrial Ca^2+^ and not mitochondrial Ca^2+^ uptake upon ER Ca^2+^ release (Figure 4J–L). We speculate that this partial enhancement of mitochondrial Ca^2+^ homeostasis is responsible for increased bioenergetics in agonist treated MCF7 cells with S1R OE and can be a reason the effect of the agonist is not as pronounced as in A549 cells, which endogenously express higher levels of S1R (Figure 1A,B and Figure 2). 

Our findings are further supported by reports showing that ligand-activated S1R increases bradykinin-induced cytosolic Ca^2+^ rise in neuroblastoma cells by dissociating adaptor protein Ankyrin from IP_3_Rs [25]. Additionally, it was shown that activation of S1R via agonist stimulation leads to the interaction of S1R with IP_3_Rs and controls the prolonged Ca^2+^ delivery to the mitochondria [1].

It is still an open question whether S1R has a constitutively active function under basal conditions. We observed mixed results upon application of S1R antagonist or S1R KD. Both S1R antagonist and KD failed to impact mitochondrial bioenergetics (Figure 2B,C and Figure 3B), which is explainable in light of unaffected mitochondrial Ca^2+^ levels (Figure 4A–F). Although it was reported that S1R stabilizes IP_3_Rs, the effect of S1R KD was mainly reported to occur upon consecutive applications of IP_3_ generating agonist [1]. Thus, it is not surprising that we have not observed an effect of S1R KD on the initial Ca^2+^ release from the ER. On the other hand, decreased cytosolic pyruvate/lactate ratio in BD1047-treated or S1R KD cells (Figure 3E,F) argues for basal activity of S1R, which might be compensated by increased aerobic glycolysis upon S1R inactivation or KD. However, the mechanism of action in this case requires further clarification.

## Data Availability

The data presented in this study are available on request from the corresponding author.

## Supplementary Materials

The following are available online at https://www.mdpi.com/article/10.3390/biom12060762/s1, Figure S1: Representative images of S1R-mCherry construct overexpressed in MCF7 cells; Figure S2: Basal mitochondrial ATP levels in control A549 (A), S1R KD A549 (B), MCF7 (C), MCF7 cells with S1R OE (D); Figure S3: Basal and 100 µM ATP induced cytosolic Ca^2+^ rise level in control A549 (A), (B), (C), S1R KD A549 (D), (E), (F), and MCF7 cells (G), (H) and (I).
