# Supplementary material for: Sigma-1 Receptor Modulation by Ligands Coordinates Cancer Cell Energy Metabolism
Source: Biomolecules. 2022 May 30;12(6):762. doi: 10.3390/biom12060762 (PMC9221035; doi:10.3390/biom12060762)
Supplement: Supplementary file 1 [file biomolecules-12-00762-s001.zip › biomolecules-1693757-supplementary.pdf]

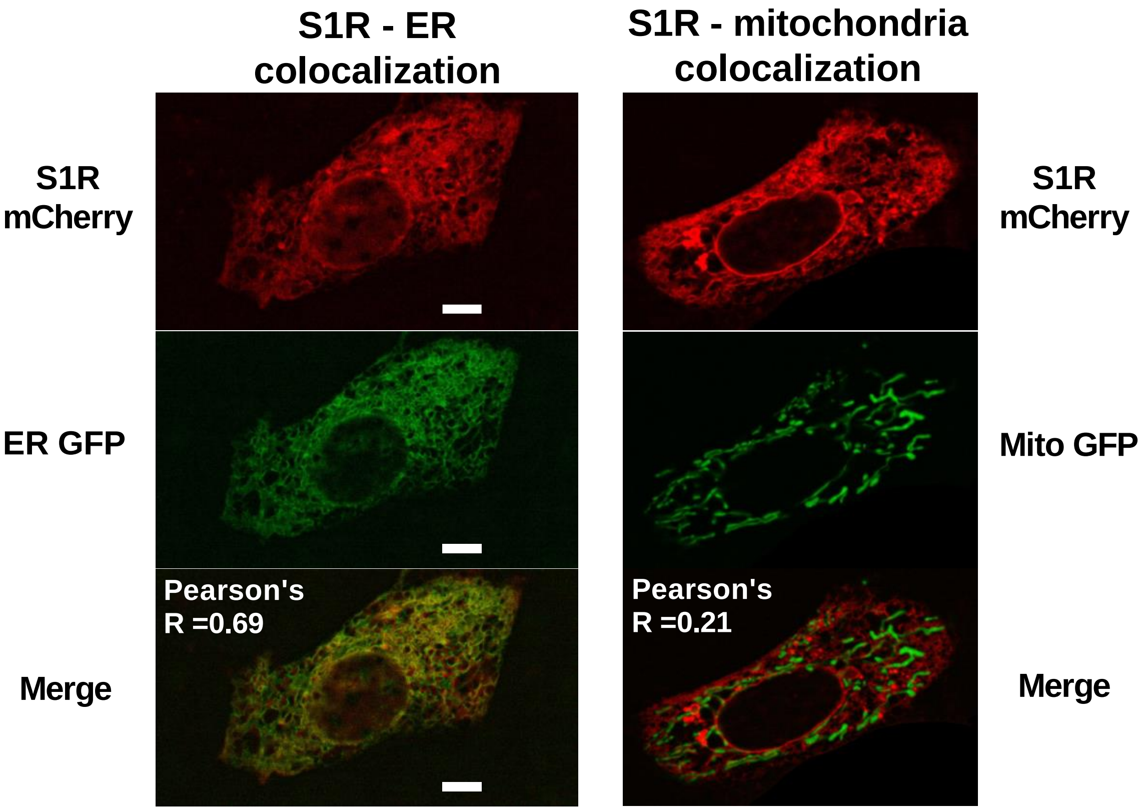

Figure S1. S1R overexpression in MCF7 cells. Overexpressed S1R-mCherry construct has a clear ER localization and colocalizes with ER-targeted GFP construct that has calreticulin targeting and ER retention sequences, and not with mitochondria targeted GFP construct.

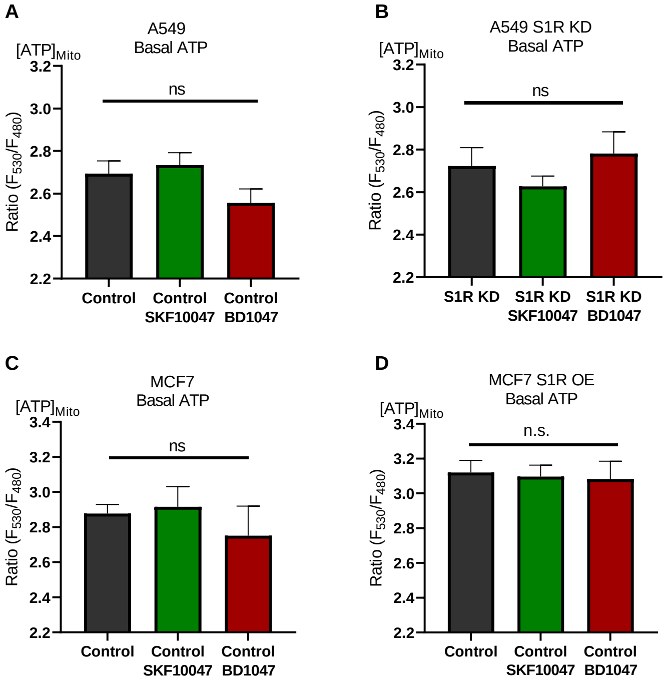

Figure S2. Effect of S1R and its ligands on mitochondrial basal ATP levels. (A) Bar graph represents the basal mitochondrial ATP levels measured with

mtAT.1.03 for control (black), Control+SKF10047 (green), Control + BD1047 (red), (B) S1R KD (black), S1R KD +SKF10047 (green) and S1R KD +BD1047 (red) in A549 cells. (C) Bar graph represents the basal mitochondrial ATP levels for control (black), Control+SKF10047 (green) and Control + BD1047 (red) in MCF7 cells. (D) Bar graph represents the basal mitochondrial ATP levels for control (black), Control+SKF10047 (green) and Control + BD1047 (red) in MCF7 cells with S1R OE. Cells were treated with BD1047 and SKF10047 2-4 h prior to each experiment. Significant differences were assessed using one-way ANOVA with Tukey's multiple comparison test and presented as (ns: not significant). A549 cells: Control (32 cells/ 12 experiments), Control + SKF10047 (29 cells/ 16 experiments), Control + BD1047 (22 cells/ 15 experiments), SR1 KD (16 cells/ 13 experiments), S1R KD + SKF10047 (15 cells/ 10 experiments) and S1RKD + BD1047 (13 cells/ 8 experiments). MCF7 cells: Control (15 cells/ 6 experiments), Control + SKF10047 (13 cells/ 4 experiments) and Control + BD1047 (11 cells/ 5 experiments). MCF7 cells with S1R OE: Control (23 cells/ 7 experiments), Control + SKF10047 (26 cells/ 8 experiments) and Control + BD1047 (18 cells/ 5 experiments).

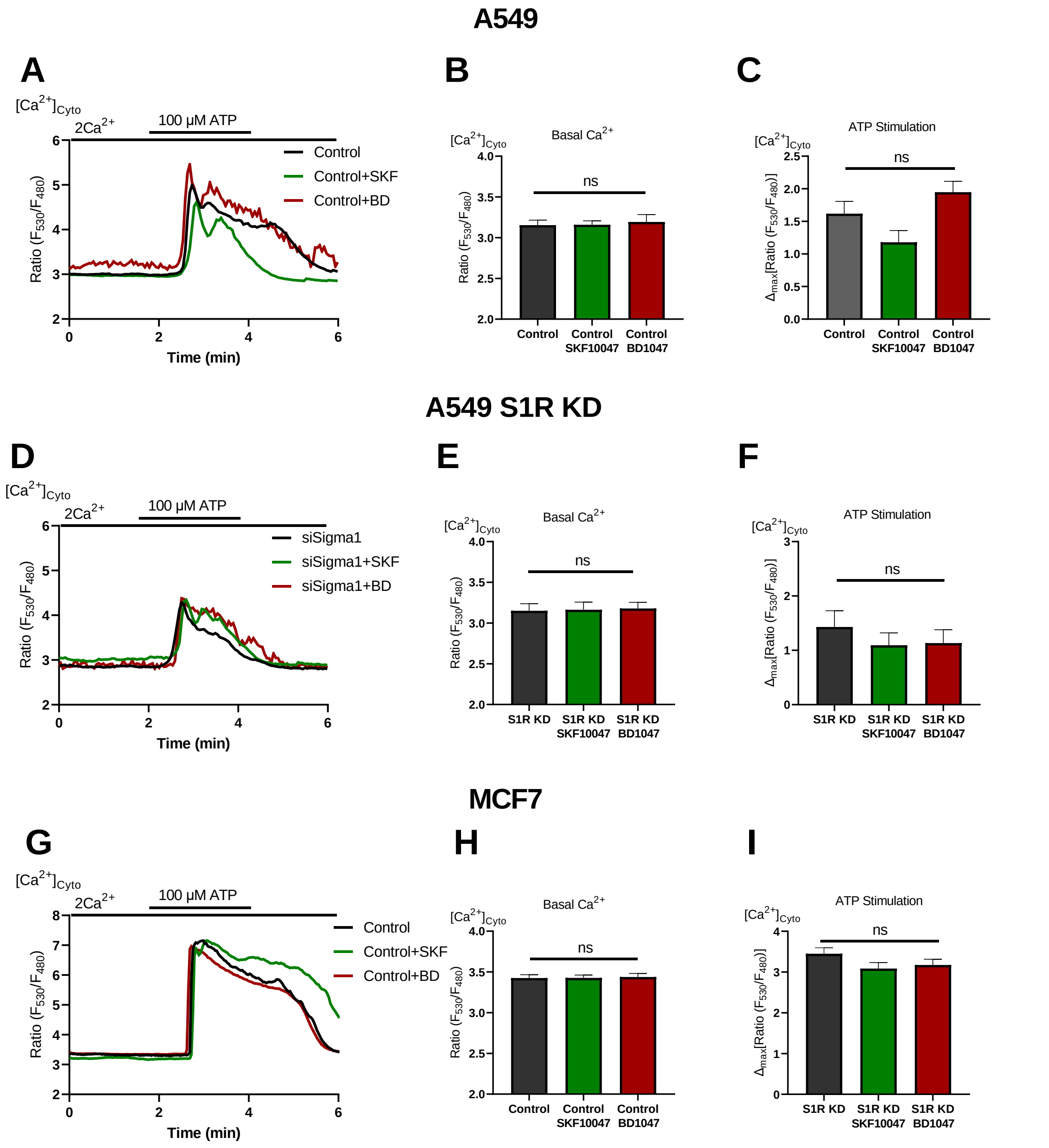

Figure S3. Effect of pharmacological activation of S1R on cytosolic Ca^{2+} homeostasis. (A) Representative traces of cytosolic Ca^{2+} levels measured with D3cpv for control (black), Control+SKF10047 (green) and Control + BD1047 (red) in A549 cells. (B) Bar graphs represents basal cytosolic Ca^{2+} level as MEAN+/-SEM for Control (black), Control+SKF10047 (green) and Control+BD1047 (red) in A549 cells. (C) Bar graphs represents ATP (100 μM) induced cytosolic Ca^{2+} uptake as MEAN+/-SEM for Control (black), Control+SKF10047 (green) and Control+BD1047 (red) in A549 cells. (D) Representative traces of cytosolic Ca^{2+} levels for S1R KD (black), S1R KD +SKF10047 (green) and S1R KD +BD1047 (red) in A549 cells. (E) Bar graphs represents basal cytosolic Ca^{2+} level as MEAN+/-SEM for S1R KD (black), S1R KD +SKF10047 (green) and S1R KD +BD1047 (red) in A549 cells. (F) Bar graphs represents ATP (100 μM) induced cytosolic Ca^{2+} uptake as MEAN+/-SEM for S1R KD (black), S1R KD +SKF10047 (green) and S1R KD +BD1047 (red) in A549 cells. (G) Representative traces of cytosolic Ca^{2+} levels for Control (black), Control+SKF10047 (green) and Control+BD1047 (red) in MCF7 cells. (H) Bar graphs represents basal cytosolic Ca^{2+} level as MEAN+/-SEM for Control (black), Control+SKF10047 (green) and Control+BD1047 (red) in MCF7 cells. (I) Bar graphs represents ATP (100 μM) induced cytosolic Ca^{2+} uptake as MEAN+/-SEM for Control (black), Control+SKF10047 (green) and Control+BD1047 (red) in MCF7 cells. Cells were treated with BD1047 and SKF10047 2-4 h prior to each experiment. Significant differences were assessed using one-way ANOVA with Tukey's multiple comparison test and presented as (ns: not significant). A549 cells basal Ca^{2+} measurements: Control (30 cells/ 6 experiments), Control + SKF10047 (33 cells/ 7 experiments), Control + BD1047 (36 cells/ 6 experiments), SR1 KD (27 cells/ 7 experiments), S1R KD + SKF10047 (29 cells/ 8 experiments) and S1RKD + BD1047 (27 cells/ 7 experiments). A549 cells ATP stimulation: Control (14 cells/ 6 experiments), Control + SKF10047 (13 cells/ 7 experiments), Control + BD1047 (21 cells/ 6 experiments), SR1 KD (14 cells/ 7 experiments), S1R KD + SKF10047 (15 cells/ 8 experiments) and S1RKD + BD1047 (12 cells/ 7 experiments). MCF7 cells basal Ca^{2+} measurements: Control (33 cells/ 3 experiments), Control + SKF10047 (44 cells/ 4 experiments) and Control + BD1047 (45 cells/ 3 experiments). MCF7 cells ATP stimulation: Control (14 cells/ 3 experiments), Control + SKF10047 (17 cells/ 4 experiments) and Control + BD1047 (18 cells/ 3 experiments).
